# Effects of Single and Combined Losartan and Tempol Treatments on Oxidative Stress, Kidney Structure and Function in Spontaneously Hypertensive Rats with Early Course of Proteinuric Nephropathy

**DOI:** 10.1371/journal.pone.0161706

**Published:** 2016-08-25

**Authors:** Danijela Karanovic, Jelica Grujic-Milanovic, Zoran Miloradovic, Milan Ivanov, Djurdjica Jovovic, Una-Jovana Vajic, Maja Zivotic, Jasmina Markovic-Lipkovski, Nevena Mihailovic-Stanojevic

**Affiliations:** 1 Department of Cardiovascular Physiology, Institute for Medical Research, University of Belgrade, 11129, Belgrade, Serbia; 2 Institute of Pathology, Faculty of Medicine, University of Belgrade, 11000, Belgrade, Serbia; Nagoya University, JAPAN

## Abstract

Oxidative stress has been widely implicated in both hypertension and chronic kidney disease (CKD). Hypertension is a major risk factor for CKD progression. In the present study we have investigated the effects of chronic single tempol (membrane-permeable radical scavenger) or losartan (angiotensin II type 1 receptor blocker) treatment, and their combination on systemic oxidative status (plasma thiobarbituric acid-reactive substances (pTBARS) production, plasma antioxidant capacity (2,2'-azino-bis(3-ethylbenzothiazoline-6-sulphonic acid, pABTS), erythrocyte antioxidant enzymes activities) and kidney oxidative stress (kTBARS, kABTS, kidney antioxidant enzymes activities), kidney function and structure in spontaneously hypertensive rats (SHR) with the early course of adriamycin-induced nephropathy. Adult SHR were divided into five groups. The control group received vehicle, while the other groups received adriamycin (2 mg/kg, i.v.) twice in a 21-day interval, followed by vehicle, losartan (L,10 mg/kg/day), tempol (T,100 mg/kg/day) or combined T+L treatment (by gavage) during a six-week period. Adriamycin significantly increased proteinuria, plasma lipid peroxidation, kidney protein oxidation, nitrite excretion, matrix metalloproteinase-1 (MMP-1) protein expression and nestin immunostaining in the kidney. Also, it decreased kidney antioxidant defense, kidney NADPH oxidase 4 (kNox4) protein expression and abolished anti-inflammatory response due to significant reduction of kidney NADPH oxidase 2 (kNox2) protein expression in SHR. All treatments reduced protein-to-creatinine ratio (marker of proteinuria), pTBARS production, kidney protein carbonylation, nitrite excretion, increased antioxidant capacity and restored kidney nestin expression similar to control. Both single treatments significantly improved systemic and kidney antioxidant defense, bioavailability of renal nitric oxide, reduced kMMP-1 protein expression and renal injury, thus retarded CKD progression. Losartan improved blood pressure, as well as tubular injury and restored anti-inflammatory defense by reverting kNox2 expression to the control level. Interestingly, tempol was more successful in reducing systemic oxidative stress, proteinuria, kMMP-1 and glomerulosclerosis. However, combined treatment failed to overcome the beneficial effects of single treatments in slowing down the progression of ADR-induced nephropathy in SHR.

## Introduction

Oxidative stress and impaired endogenous antioxidant defense are associated with hypertension [1] and chronic kidney disease (CKD) [2]. Hypertension, *per se*, is the second most common cause of CKD and represents the primary risk factor of its progression [3]. Spontaneously hypertensive rats (SHR) represent a widely accepted animal model for essential hypertension, and in advanced age they develop all pathophysiological and clinical alterations resembling CKD in patients [4]. Moreover, SHR that receive adriamycin (ADR, doxorubicin hydrochloride) develop focal segmental glomerulosclerosis [3,5] followed by massive proteinuria (a nephrotic syndrome severity marker), which is tightly associated with the loss of kidney function [3]. Further, increased production of reactive oxygen species (ROS) and other free radicals are also involved in doxorubicin action [6]. Adriamycin leads to direct oxidative injuries of DNA, generates lipid peroxidation [6], induces protein oxidation [7], and impairs endogenous antioxidant defense [2]. Endogenous defense system involved in neutralization of ROS includes antioxidant enzymes such as superoxide dismutase (SOD), catalase (CAT), and glutathione peroxidase (GSH-Px).

Angiotensin II (AngII) participates in subcellular mechanisms and signaling pathways that contribute to oxidative stress due to AngII type 1 receptor (AT1R) stimulated NAD(P)H oxidase activity, the major source of superoxide anion (^.^O_2_^-^) production in hypertension [1]. In addition, AngII plays an important role in CKD progression and its effects may be blocked either by angiotensin converting enzyme inhibitors (ACEI) or AT1R antagonists [8]. Previously, it has been shown that captopril significantly decreased systolic blood pressure, but failed to prevent proteinuria in ADR-treated SHR [5]. Further, in the same experimental model losartan, beside the antihypertensive effect, improved kidney function due to decrease of proteinuria, which still remained significantly higher compared to non-proteinuric SHR [3]. Thus, these observations confirmed the importance of AngII AT1R-mediated non-haemodynamic actions in slowing down the CKD progression [3].

Synthetic antioxidant, tempol (4-hydroxy-2,2,6,6-tetramethylpiperidine-N-oxyl) is a cell membrane-permeable nitroxide that mimics the enzyme superoxide dismutase to reduce^.^O_2_^-^ (SOD mimetic), or can be converted into hydroxylamines or oxoammonium to directly scavenge free radicals [9]. Tempol efficiently decreased blood pressure in various animal models of hypertension [9] and improved kidney function in Swiss albino mice with cisplatin-induced nephrotoxicity, streptozotocin-induced diabetic rats, and obese Zucker rats—a model of type 2 diabetes and metabolic syndrome [10–12], but its role in SHR with ADR-induced nephropathy remains unclear.

There are also other cellular sources of free radicals that can contribute to their overproduction, which include mitochondrial disruption, xanthine oxidase, and uncoupled endothelial nitric oxide (NO) synthase [1]. Therefore, blocking both AT1R-dependent and–independent free radical production could be more beneficial therapeutic approach. It has been previously shown that various antioxidants can be incorporated into the biphenyl sartan scaffold to form a dual action drug [13]. Jani et al. [13] demonstrated that the incorporation of nitroxide into milfsartan yielded the drug, nitrasartan, which retained antioxidant and AT1R antagonist activity when used *in vitro* assay. The data published by Dobrian et al. [14] showed a synergistic effect of AT1R antagonist, losartan and nitroxide, tempol on blood pressure reduction in one-kidney, one-clip model of renovascular hypertension. However, to our knowledge there are no studies related to the effects of prolonged combined application of tempol and losartan on progression of ADR-induced nephropathy in SHR.

Thus, the aim of the present study was to examine the effects of single chronic tempol (membrane-permeable antioxidant), and losartan (AT1R), or their combined treatment on oxidative stress, antioxidant defense system, NO content, kidney function and structure of SHR with the early stage of ADR-induced proteinuric nephropathy.

## Materials and Methods

### 1. Animals

Adult SHR females (six-month-old, 180–200 g body weight (b.w.)) were housed under standard conditions of humidity and temperature with a 12h light/dark cycle (Institute for Medical Research, Belgrade, Serbia). Standard food (Veterinarski zavod Subotica, Serbia) and tap water were provided *ad libitum*. The experimental protocol was in accordance with the National Law on Animal Welfare (“Sl.gl.RS” No6/10) and approved by the Ethic Committee of the Institute for Medical Research, University of Belgrade, Serbia and Veterinary Directorate, Minister of Agriculture and Environmental Protection, Republic of Serbia (No323-07-00318/2015-05). All surgeries were performed under sodium pentobarbital anesthesia, and all efforts were made to minimize suffering.

### 2. Experimental Protocol

The rats were randomly divided into five groups according to previously non-invasive (Physiograph Four, Narco Bio-system Houston, TX, USA) systolic blood pressure measurement. Four experimental groups received ADR (2 mg/kg b.w.) twice in a 21-day interval, intravenously (femoral vein) under anesthesia (sodium pentobarbital, 35 mg/kg b.w., intraperitoneally (i.p.)). The control group (SHC) was injected with a comparable volume of 0.9% saline. After the second injection of saline or ADR, SHC and SHADR received tap water, while SHADR+L, SHADR+T, and SHADR+T+L groups received losartan (L, 10 mg/kg/day b.w., Dup 753, DuPont, Wilmington De.), tempol (T, 100 mg/kg/day b.w., Sigma-Aldrich), or both (T+L) by gavage during the next six weeks, respectively. At the end of the study, rats were placed in individual metabolic cages for a 24-hour urine collection. After this period, haemodynamic measurements, followed by blood sample and kidney collection were performed on anaesthetized animals.

### 3. Haemodynamic Parameter Determination

Systolic arterial pressure (SAP) was measured in anesthetized rats (sodium pentobarbital, 35 mg/kg b.w. i.p.) directly through a femoral artery catheter (PE–50, Clay-Adams Parsippany, NY, USA) connected to a physiological data acquisition system (9800TCR Cardiomax III-TCR Thermodilution Cardiac Output, Columbus, OH, USA).

The segment of abdominal aorta above bifurcation of left renal artery was separated from surrounding tissue, and an ultrasonic flow probe (2RB, internal diameter 2 mm) was placed around the aorta. Aortal blood flow (ABF) was recorded by Transonic T106 Small Animal Flowmeter (Transonic System Inc., Ithaca, NY, USA).

### 4. Sample Collection

Blood samples were obtained by abdominal aorta puncture, using an anticoagulant-EDTA (Ethylenediaminetetraacetic acid disodium salt dehydrate, Division of ICN Biomedicals, Inc.Cleveland, Ohio), and centrifuged at 4000 rpm, 4°C for 20 min. Plasma was collected and stored at -20°C until assaying. The remaining erythrocyte pellet was resuspended in ice-cold saline and centrifuged four times at 2000 rpm, 4°C for 10 min, and stored at -70°C until assaying. Kidneys were removed, rinsed in ice-cold saline, and weighed. The tissue was immediately frozen in liquid nitrogen, and stored at −70°C for later analysis.

### 5. Biochemical Measurements

Plasma creatinine concentration (Pcr), as well as protein (Up) and creatinine levels in 24h urine were measured by an automatic COBAS INTEGRA 400 plus analyzer (Hoffmann-La Roche, Leitch Diagnostic, Germany). Urine protein-to-creatinine ratio (Up/cr) and Up were used for the assessment of proteinuria.

### 6. Measurements of Oxidative Stress Markers

For the assessment of lipid peroxidation, thiobarbituric acid-reactive substances (TBARS) were estimated by using 2-thiobarbituric acid (2,6-dihydrooxy pyrimidine-2-thiol, Acros, Organic) [15] in plasma, urine, and kidney homogenates. Plasma TBARS values were normalized to abdominal aorta blood flow to evaluate the rate of TBARS production, and expressed as nmol/min/kg. An extinction coefficient 156000 M^-1^cm^-1^ was used for calculation. Values were expressed as nmol/min/kg for urine, and nmol/mg tissue for kidney samples. Total antioxidant status, the capacity of plasma for free radicals neutralization, was measured using 2,2’-azinobis-(3-ethylbenzothiazoline-6-sulfonic acid) (ABTS^•+^) radical formation kinetics. The presence of antioxidants in plasma suppressed the bluish-green staining of the ABTS cation, which was proportional to the antioxidant concentration level. Kinetics was measured at 734 nm as previously described [16]. The data are expressed as mmol Trolox Equivalents (TE)/l of plasma. The ABTS assay was also performed in kidney homogenates, and values are expressed as nmol TE/g of tissue.

Protein carbonyl content (PCOs), a general indicator of protein oxidation, was quantified in the kidney by reaction with 2,4-dinitrophenylhydrazine (2,4-DNPH), as previously shown [17]. Total PCOs were determined from the absorbance at 370 nm using a 2,4-DNPH molar absorption coefficient of 0.022 μM^-1^cm^-1^, and expressed as nmol/mg protein.

### 7. Antioxidant Enzyme Activity

The activities of antioxidant enzymes (SOD, CAT, and GSH-Px) were measured in erythrocytes and kidney homogenates by the spectrophotometric method, as previously described [18–20].

### 8. Quantitative Sandwich Enzyme-Linked Immunodetection of Antioxidant Enzymes, MMP-1, and Nox4 Protein

Erythrocyte SOD1, CAT, and GSH-Px expressions were determined using quantitative rat-specific ELISA kits (Cusabio, Wuhan, Hubei, China). Previously frozen erythrocytes were processed in accordance with the manufacturer's recommendations. Briefly, erythrocytes were dissolved with ice-cold deionized water, and stored at -20°C for 30 min. After three freeze-thaw cycles to break up the cell membranes, lysates were centrifuged at 10000 rpm, 4°C for 10 min. The supernatant was further assayed, and values were expressed as pg/ml for SOD1, and mIU/ml for CAT and GSH-Px.

Quantitative determinations of Nox4 and MMP-1 in kidney homogenate were performed by rat-specific ELISA kits (Nox4: Cloud-Clone Corp., Houston, USA; MMP-1: BlueGene Biotech, Shanghai, China) according to the instruction, and data are expressed as ng/ml.

### 9. Western Blot Analysis for NADPH oxidase Subunit Nox2 Protein

The kidneys were homogenized in chilled RIPA lysis buffer (50 mM Tris-HCl pH 7.5, 150 mM NaCl, 1% Triton x-100, 1% sodium deoxycholate, 0.1% sodium dodecyl sulphate, 2 mM EDTA, and 50 mM NaF) at a ratio of 100 mg of tissue to 1 ml of buffer. A protease inhibitor cocktail (Pierce, Thermo Fisher Scientific, Waltham, MA, USA) and sodium orthovanadate were added to the lysis buffer prior to use. Lysates were incubated at 4°C for 20 min, and then centrifuged at 15,000 x g, 4°C, for 20 min. Protein concentration was determined by the BCA Protein Assay Kit (Pierce, Thermo Fisher Scientific) and samples were stored at -70°C until analysis.

For Western blotting, equal amounts of protein samples were run on 10% polyacrylamide gels and transferred to nitrocellulose membranes (Appli-Chem GmbH, Darmstadt, Germany). Membranes were probed with primary antibodies to Nox2 (1:500, Abcam, ab31092), and actin (1:1000, Sigma-Aldrich, A5060). Peroxidase-conjugated goat anti-rabbit immunoglobulin (1:40.000, Sigma-Aldrich, A0545) was used as a secondary antibody. Western blots were developed using the enhanced chemiluminescence reagent system (GE Healthcare, Amersham, UK) according to the manufacturer’s instructions. The content of Nox2 in the tissue extracts was estimated by the densitometry of scanned immunoblot bands using the Image Master Total Lab (GE Healthcare) software.

### 10. Measurements of NO metabolites (NO_x_): NO_2_ and NO_3_

Concentration of NO_x_ (total NO_2_ and NO_3_-stable oxidation products of NO) was measured in urine and kidney homogenates by the Griess reagent method, as previously described [20]. Values for urinary NO_2_ and NO_3_ excretions were expressed as nmol/24h, and for the NO_x_ content in the kidney homogenates as μmol/mg tissue.

### 11. Histological Examination

For light microscopy observation, kidney sections were fixed in 10% buffered formalin, dehydrated in alcohol, embedded in paraffin, cut into 5 μm slices, and stained with periodic acid-Schiff reagent (PAS). Slides were examined at magnification x20 by pathologist blind to the experimental profile. Sclerotic changes in glomeruli were graded as follows: 0 normal glomeruli, 1+ slight segmental change in small number of glomeruli, 2+ segmental and global changes in most glomeruli, and 3+ general global sclerosis. Tubular dilatation with luminal PAS positive material and atrophy of tubular epithelium were graded from 0 to 3+ according to the degree of lesions; the sum of these changes is presented as index of tubular damage. Interstitial infiltration and fibrosis were graded from 0 to 3+ according to the extension of changes. The renal injury score represents the sum of observed changes for comparison between groups.

### 12. Immunoperoxidase staining

Immunostaining was applied on 5 μm thick paraffin sections. After deparaffinization and rehydration, the sections were treated by microwave for 20 min at 400 W in citrate buffer (pH 6.0). After antigen retrieval, samples were incubated for 1 hour at room temperature with primary antibody for nestin (Santa Cruz, USA, sc-23927, dilution 1:100). Sections were then treated with EnVisionTM Detection System (DAKO, Germany) using 3-amino-9-ethylcarbazole (AEC) as substrate, and counterstained with hematoxylin. Negative controls were performed by omitting the first antibody and stained by the EnVisionTM method, and for mouse monoclonal antibodies as isotype control mouse IgG1 (ab91353, Abcam, UK) antibody was also used. The slides were evaluated using a light microscope BX53 with DP12-CCD camera (Olympus, Germany).

### 13. Statistical Analysis

The data are presented as mean ± SEM. One-way analysis of variance (ANOVA) was used for multiple comparisons between experimental groups. Fisher LSD test was performed as a *post hoc* multiple comparison test (Statistica 8). The Pearson correlation between the examined parameters was also determined. *P*-value *<*0.05 was considered significant.

## Results

### 1. Blood Pressure and Biochemical Measurements

**Fig 1:**
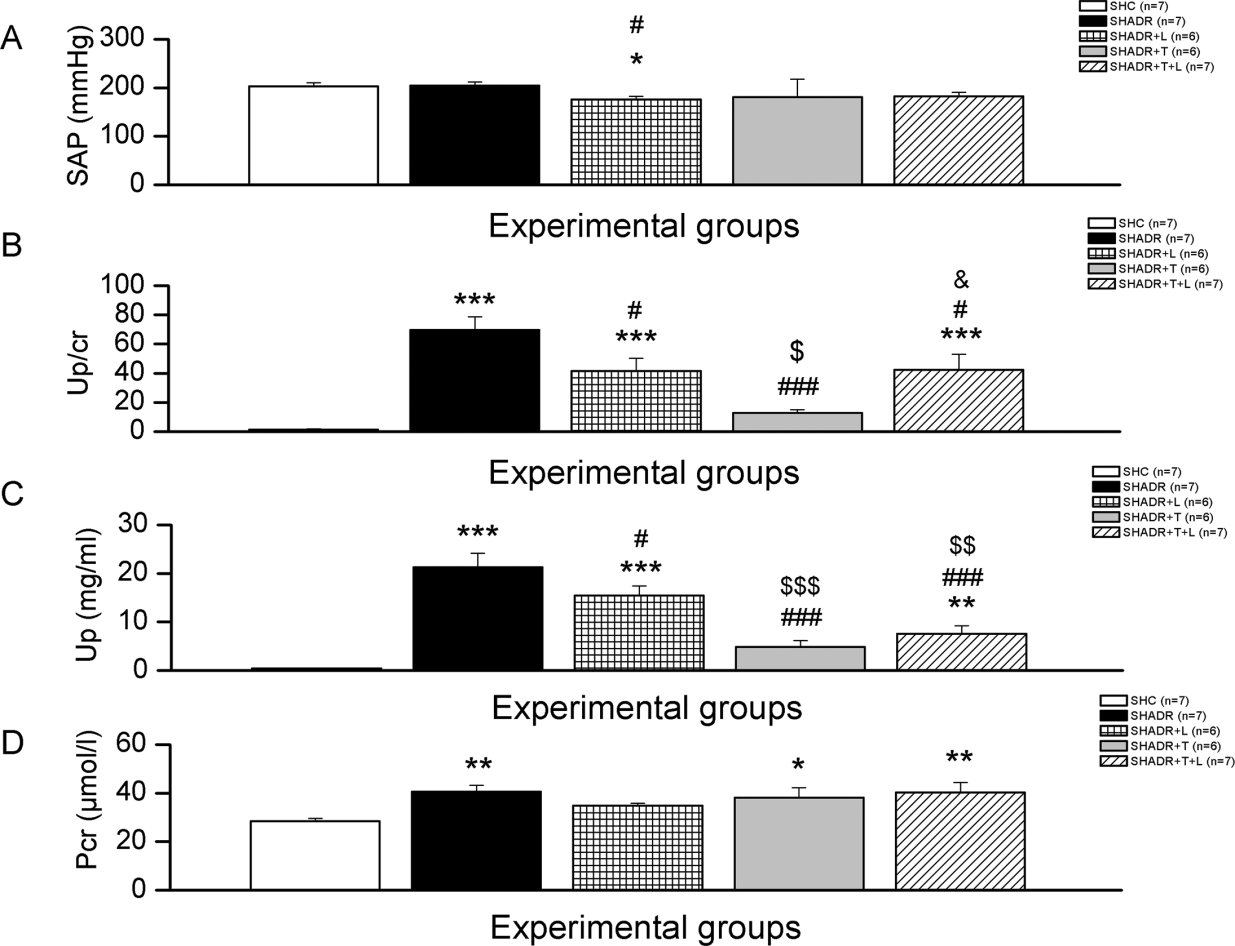
Systolic arterial pressure (SAP), urine protein-to-creatinine ratio (Up/cr), urine protein (Up), and plasma creatinine (Pcr) in experimental groups. *p<0.*05*, **p<0.*01*, ***p<0.*001* vs. SHC; *#p<0*.*05*, *###p<0*.*001* vs. SHADR; *$p<0*.*05*, *$ $p<0*.*01*, *$ $ $p<0*.*001* vs. SHADR+L; n = 6–7 animals per group. Data represent mean ± SEM. SHC—control group, SHADR–SHR treated with adriamycin, L—losartan, T–tempol.

The SAP value of SHADR group was similar to control (Fig 1A). Losartan significantly decreased SAP and tempol slightly lowered this value (~12%) compared to SHADR group. Combined treatment lowered SAP value (~11%) and brought it near the SHADR+T group.

Adriamycin in a cumulative dose of 4 mg/kg induced massive proteinuria (Fig 1B). Losartan, similar to combined therapy, significantly decreased proteinuria, which still remained higher than in control. However, tempol therapy was more effective than both, losartan and combined treatment in decreasing proteinuria in ADR-treated SHR.

Urine protein level was significantly increased in SHADR compared to control group (Fig 1C). All treatments significantly decreased this protein loss, but tempol alone or in combination with losartan induced greater reduction of Up compared to SHADR group.

Significant increase of plasma creatinine concentration was observed in SHR after adriamycin application at the end of the 6^th^ week of experiment (Fig 1D). Losartan treatment lowered this value to the level not significantly different from control. Tempol and combined therapy showed no further change in Pcr of SHADR group.

### 2. Antioxidant Enzymes

**Fig 2:**
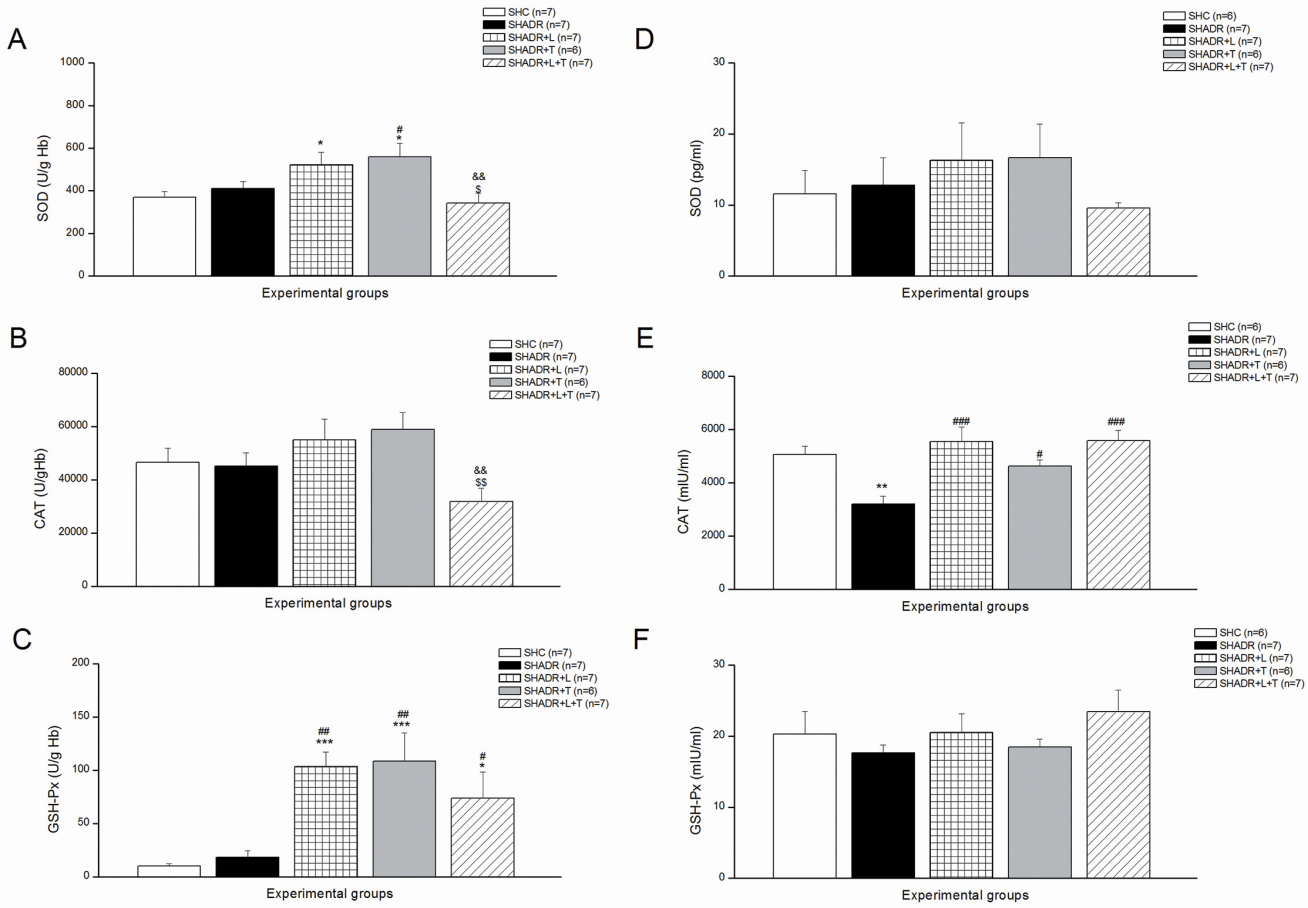
**Antioxidant enzymes activities (A-C) and expressions (D-F) in erythrocytes among the experimental groups.** SOD—superoxide dismutase, CAT—catalase, and GSH-Px—glutathione peroxidase. *p<0.*05*, **p<0.*01*, ***p<0.*001* vs. SHC; ^#^*p<0*.*05*, ^##^*p<0*.*01*, ^###^*p<0*.*001* vs. SHADR; ^$^*p<0*.*05*, ^$ $^*p<0*.*01* vs. SHADR+L; ^&&^*p<0*.*01* vs. SHADR+T; n = 6–7 animals per group. Data represent mean ± SEM. SHC—control group, SHADR–SHR treated with adriamycin, L—losartan, T—tempol.

Erythrocyte SOD, CAT, and GSH-Px activities and expressions are shown in Fig 2. Adriamycin caused no additional alterations in activity of antioxidant enzymes in SHR. Single chronic treatments with losartan and tempol significantly increased SOD and GSH-Px activities. However, combined treatment significantly decreased SOD and CAT activities compared to single therapies, while slightly lowered GSH-Px activity was still significantly higher than in control. The expression of CAT was significantly reduced in SHADR compared to control, and all treatments reverted CAT expression near to control level. SOD and GSH-Px expressions remained unchanged in this study.

**Fig 3:**
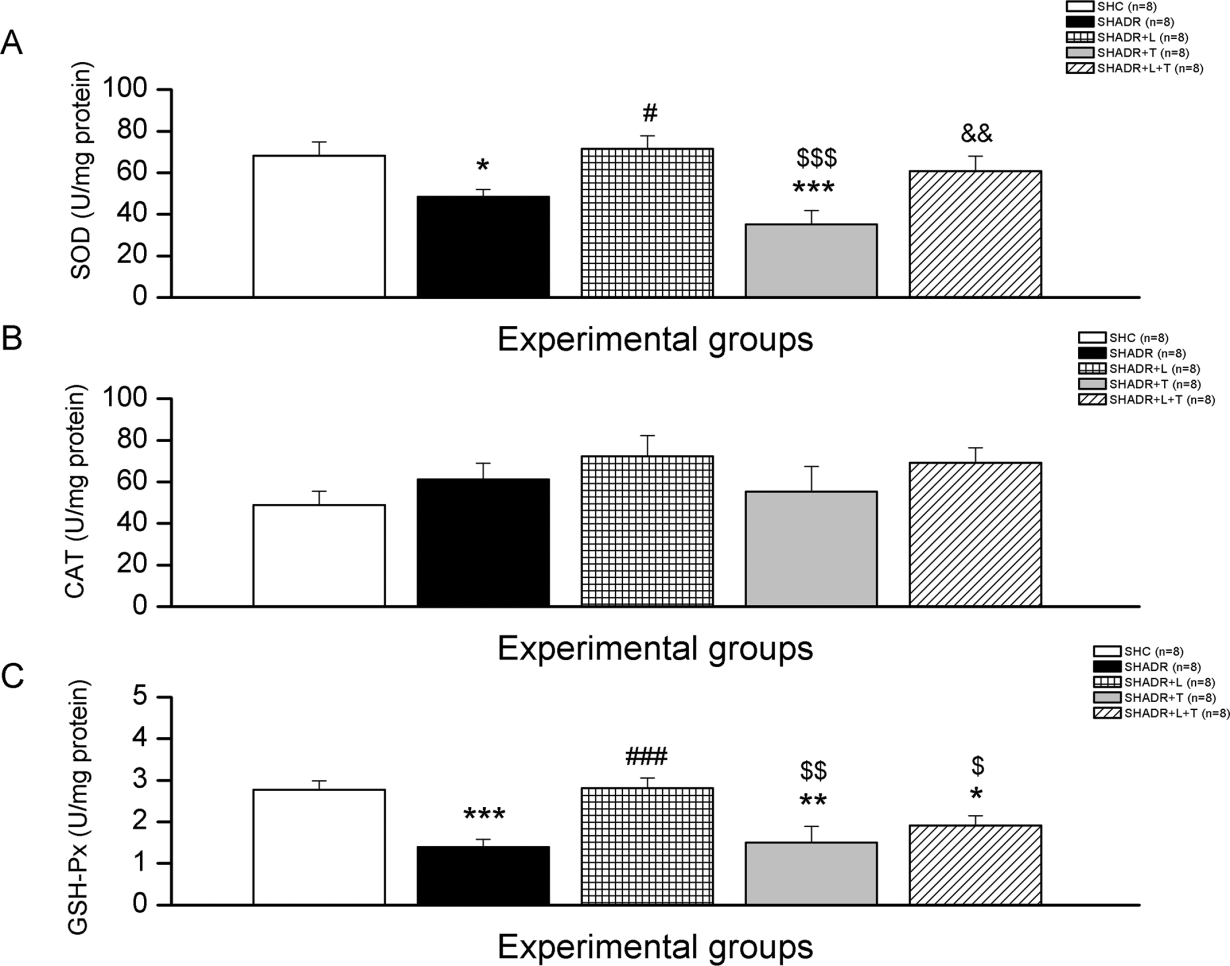
Antioxidant enzymes activities in kidney among the experimental groups. SOD—superoxide dismutase, CAT—catalase, and GSH-Px—glutathione peroxidase. *p<0.*05*, **p<0.*01*, ***p<0.*001* vs. SHC; ^#^*p<0*.*05*, ^###^*p<0*.*001* vs. SHADR; ^$^*p<0*.*05*, ^$ $^*p<0*.*01*, ^$ $ $^*p<0*.*001* vs. SHADR+L; ^&&^*p<0*.*01* vs. SHADR+T; n = 8 animals per group. Data represent mean ± SEM. SHC—control group, SHADR–SHR treated with adriamycin, L—losartan, T—tempol.

Antioxidant enzymes activities of SOD and GSH-Px in kidney were significantly decreased in ADR-treated SHR (Fig 3). Losartan treatment significantly increased these activities and reverted them to control levels. However, tempol showed no additional changes to SOD and GSH-Px activities compared to SHADR group. Contrary to single treatment, tempol in combination with losartan induced significant increase of SOD activity, bringing it closer to control, and it was not significantly different from SHADR+L. However, the GSH-Px activity was similar in SHADR+T+L, SHADR+T and SHADR, but still significantly reduced in SHADR+T+L compared to control. The CAT activity in kidney remained unchanged in this study.

### 3. Markers of Oxidative Stress

**Fig 4:**
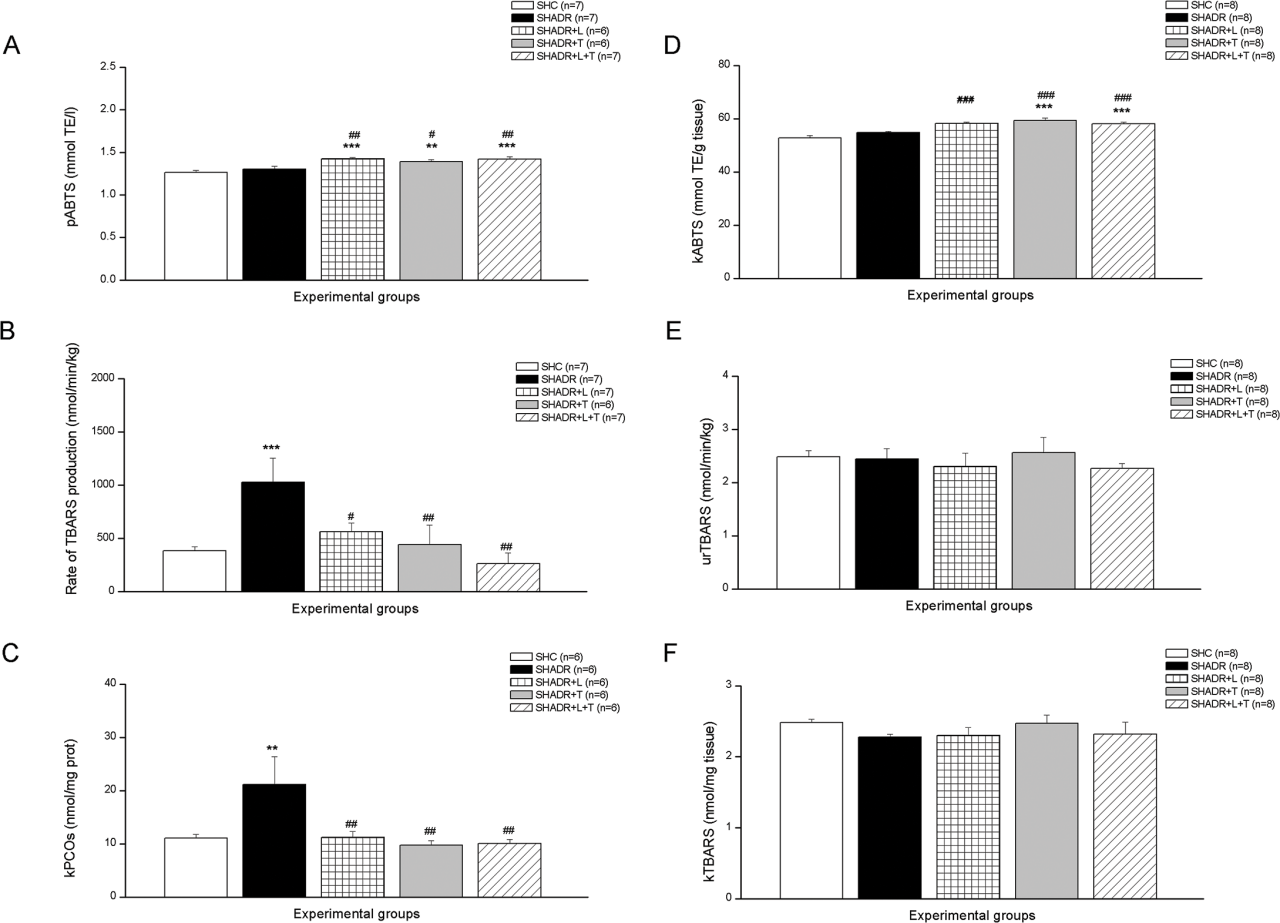
**Lipid peroxidation (TBARS) (B, E, and F), antioxidant capacity (ABTS) (A, D), and protein oxidation (PCOs) (C) among the experimental groups.** **p<0.*01*, ***p<0.*001* vs. SHC; *#p<0*.*05*, *##p<0*.*01*, *###p<0*.*001* vs. SHADR; p–plasma, k–kidney, ur–urine, n = 6–8 animals per group. Data represent mean ± SEM. SHC—control group, SHADR–SHR treated with adriamycin, L—losartan, T—tempol.

The plasma ABTS level in control was not changed compared to SHADR group (Fig 4A). However, plasma antioxidant capacity was significantly increased after all treatments. Similar observations were also found in the kidney regarding ABTS levels (Fig 4D).

The rate of TBARS production was significantly increased in SHADR group compared to control, and all treatments significantly reduced this value compared to SHADR (Fig 4B). There were no significant differences among the experimental groups regarding TBARS levels in the urine and kidney (Fig 4E and 4F).

Adriamycin in a cumulative dose of 4 mg/kg induced significant increase of PCOs in kidney in SHR (Fig 4C). This high level of protein oxidation was significantly decreased following all treatments compared to SHADR group, and it reached the same level as in control.

### 4. NO_x_, NO_2_ and NO_3_ Levels

**Fig 5:**
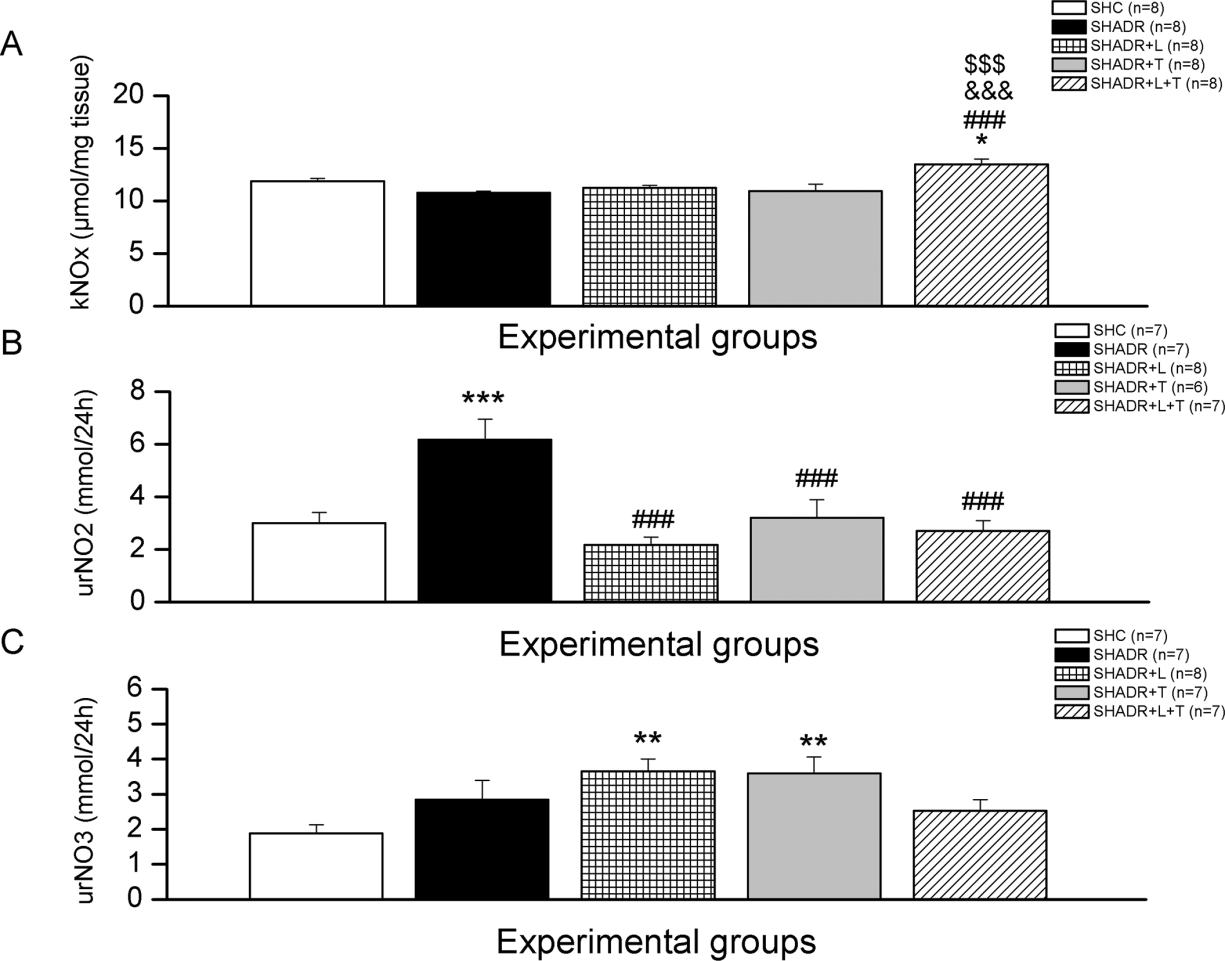
**Renal NOx content (A), urinary NO**_**2**_ **and NO**_**3**_ **excretions (B, C) in experimental groups.** *p<0.*05*, **p<0.*01*, ***p<0.*001*vs. SHC; *### p<0*.*001* vs. SHADR; *$ $ $ p<0*.*001* vs. SHADR+L; *&&& p<0*.*001* vs. SHADR+T; k–kidney, ur–urine, n = 6–8 per group. Data represent mean ± SEM. SHC—control group, SHADR–SHR treated with adriamycin, L—losartan, T—tempol.

The kidney level of NO_x_ in ADR-treated SHR was similar to control (Fig 5A). Both single therapies did not change this value, but combined treatment induced significant increase of NO_x_ level in the kidney compared to all experimental groups. The urine excretion of NO_3_ was not changed, but NO_2_ was significantly increased in SHADR group compared to control (Fig 5C and 5B). All chronic treatments significantly reduced NO_2_ excretion in SHADR, but NO_3_ excretion significantly increased following only single treatments compared to control.

### 5. Structural Alterations

**Fig 6:**
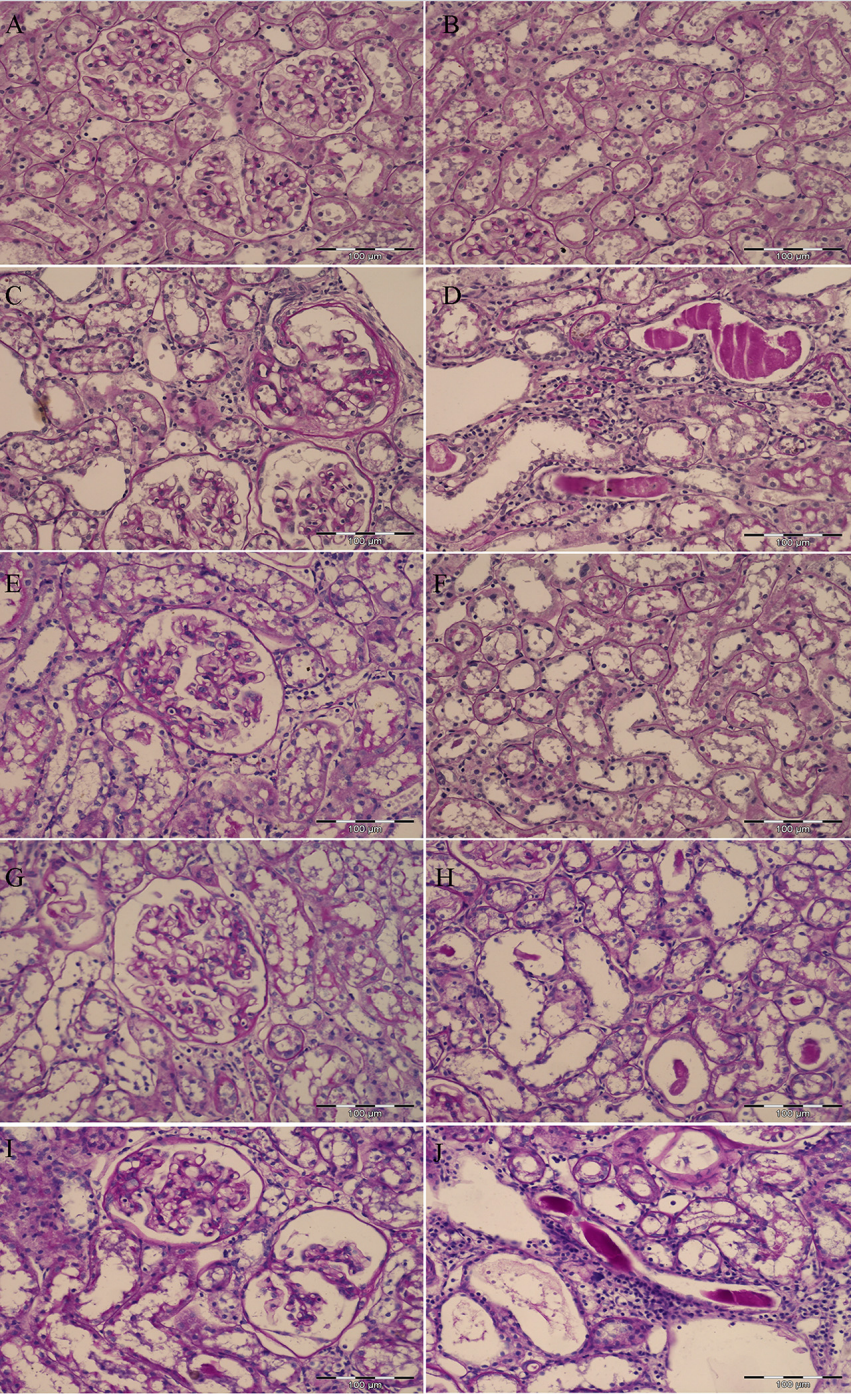
Morphological changes of glomeruli and tubules in experimental groups (PAS, x20 magnification, bar = 100μm). (**A**) Normal shape of glomeruli and (**B**) tubulointerstitium in SHC group. (**C**) Glomerulus (upper right) with advanced glomerular sclerosis involving almost entire glomerular tuft, periglomerular interstitial fibrosis and mononuclear inflammatory infiltrate; (**D**) tubular atrophy and dilatation with PAS positive casts, interstitial fibrosis with mononuclear inflammatory infiltrate in SHADR group. (**E**) Moderate capsular adhesion and segmental glomerular sclerosis involving almost one third of the glomerular tuft; (**F**) well-preserved tubules and interstitium in SHADR+L group. (**G**) Glomerulus with mild capsular adhesions and incipient sclerosis; (**H**) slightly prominent tubular dilatation with infrequent PAS positive casts in SHADR+T group. (**I**) Left glomerulus with capsular adhesion and advanced segmental glomerular sclerosis, right glomerulus with capsular adhesion, incipient segmental sclerosis, and collapse of the glomerular capillary loops; (**J**) tubular dilatations with PAS positive casts, interstitial mononuclear inflammatory infiltrate, and slight interstitial fibrosis in SHADR+T+L group.

Morphological changes in the kidney of all experimental groups are shown in Fig 6. Regular glomerular and tubulointerstitial morphology were found in control SHR group (Fig 6A and 6B). An advanced glomerular sclerosis with capsular adhesion of the glomerular tuft and the presence of periglomerular mononuclear inflammatory infiltrate with interstitial fibrosis were observed in ADR-treated SHR (Fig 6C). An advanced, chronic tubulointerstitial lesions including tubular atrophy and dilatation with PAS positive casts, and interstitial fibrosis with mononuclear inflammatory infiltrate were also present in the early phase of ADR-induced nephropathy (Fig 6D). Moderate glomerular changes with morphologically well-preserved tubulointerstitial compartment were observed after chronic losartan treatment (Fig 6E and 6F). Mild glomerular changes with slight capsular adhesions and incipient sclerosis followed by moderate tubular affection were present after prolonged tempol treatment (Fig 6G and 6H). Kidneys after combined losartan and tempol treatment exhibited an advanced segmental glomerular sclerosis with capsular adhesion, and similar degree of tubulointerstitial lesions as in SHADR group (Fig 6I and 6J).

**Fig 7:**
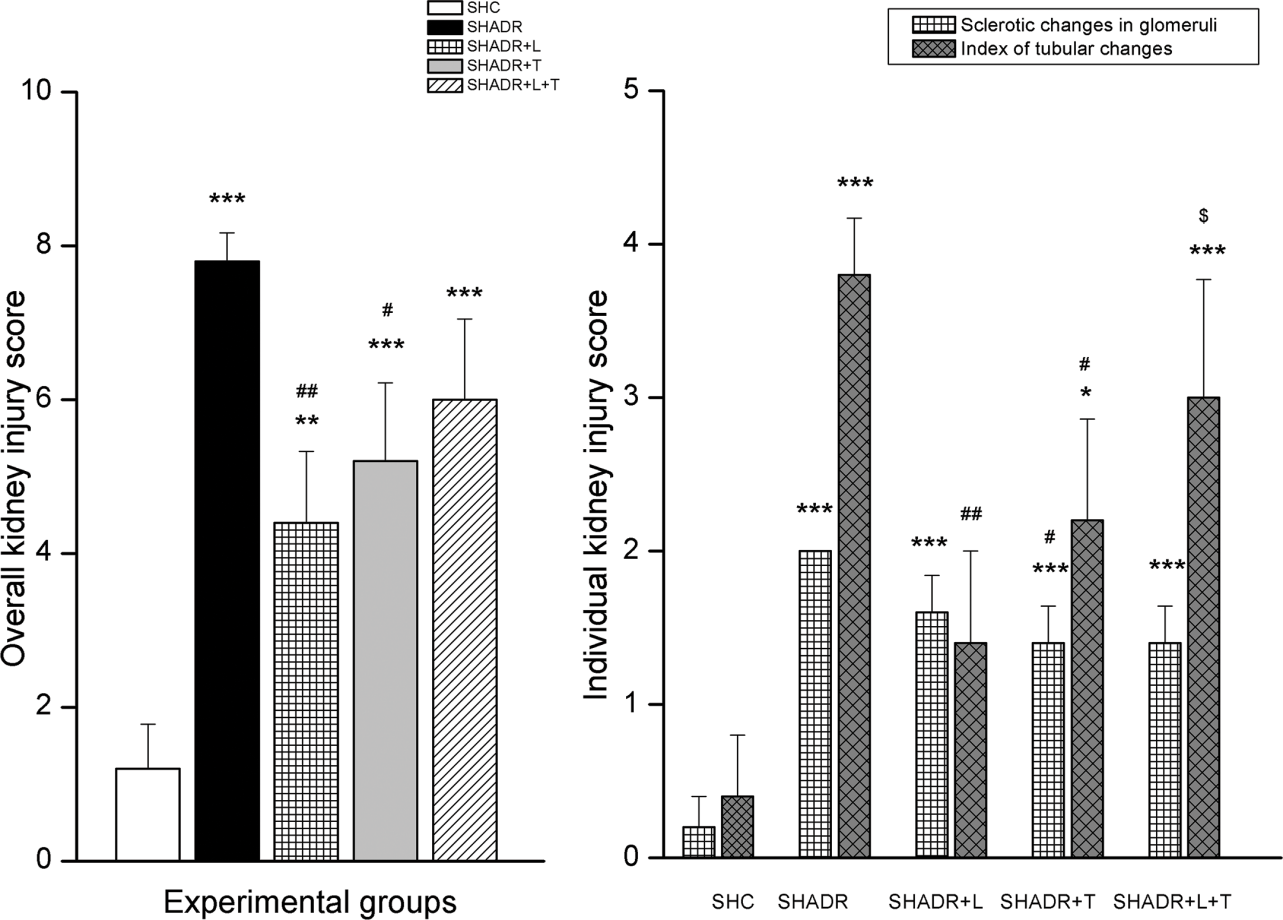
**(A) Overall and (B) individual scores of glomerular and tubular injury in experimental groups.** *p<0.*05*, **p<0.*01*, ***p<0.*001* vs. SHC; *#p<0*.*05*, *##p<0*.*01* vs. SHADR; *$p<0*.*05* vs. SHADR+L; Data represent mean ± SEM; n = 6 rats in each group; SHC—control group, SHADR–SHR treated with adriamycin, L—losartan, T—tempol.

The renal injury score (Fig 7) demonstrates that glomerulosclerosis and tubular injuries were significantly increased in ADR-treated SHR compared to control SHR. Both single therapies significantly reduced these alterations in the kidney, but more pronounced effect was observed after losartan treatment. On the other hand, losartan and tempol in combination showed no significant amelioration of structural changes in the early course of ADR-induced nephropathy in SHR.

### 6. MMP-1, Nox2 and Nox4 Protein Expression

**Fig 8:**
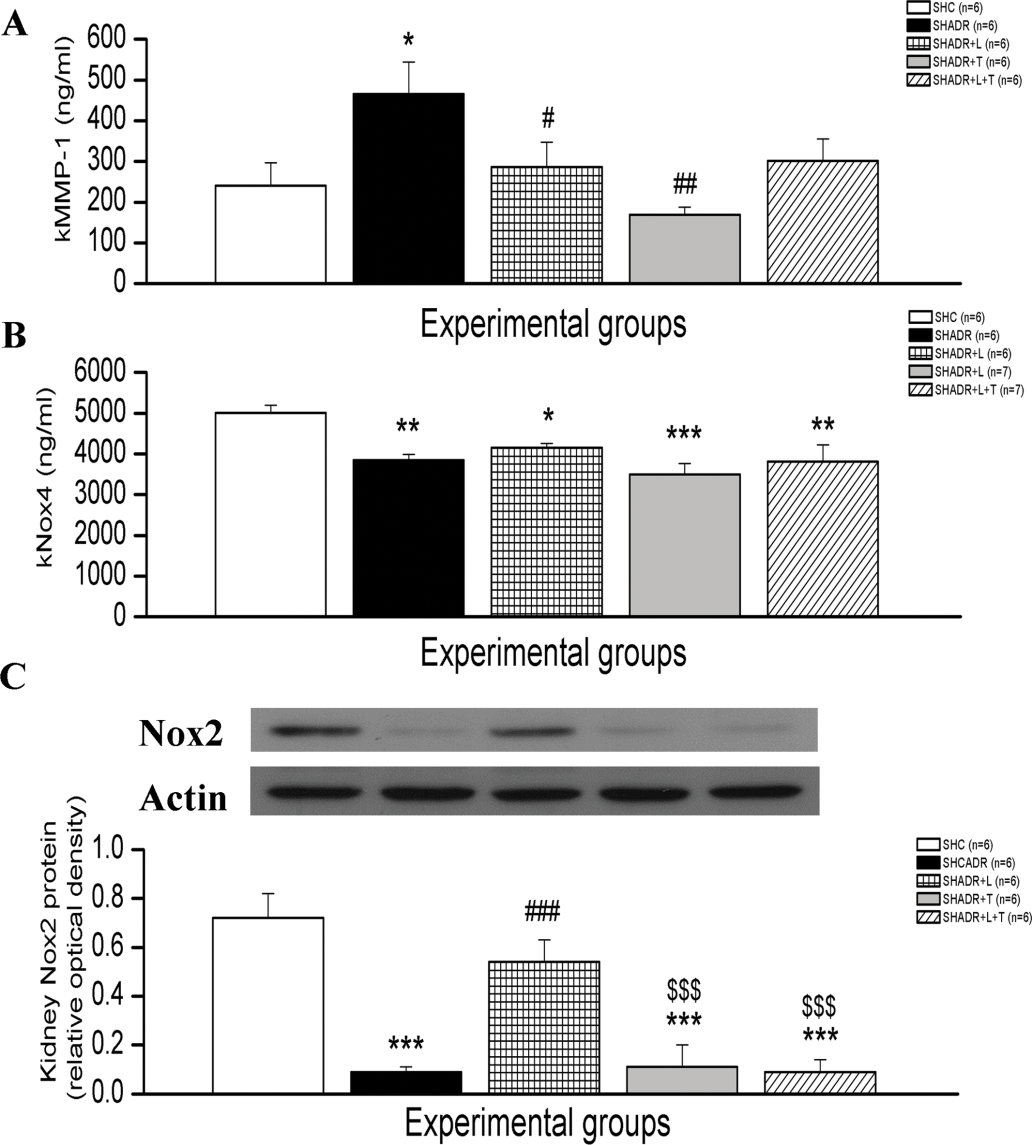
Kidney MMP-1, Nox4 and Nox2 protein levels in experimental groups. *p<0.*05*, **p<0.*01*, ***p<0.*001* vs. SHC; *###p<0*.*001* vs. SHADR; *$ $ $p<0*.*001* vs. SHADR+L; k- kidney; Data represent mean ± SEM; n = 6 rats in each group; SHC—control group, SHADR–SHR treated with adriamycin, L—losartan, T–tempol.

MMP-1 protein expression was significantly elevated in SHADR group compared to SHC, and single treatments with losartan or tempol markedly reduced its expression to value as in SHC (Fig 8A). Significant reduction of Nox2 and Nox4 protein levels were observed in kidney of SHADR compared to control (Fig 8B and 8C). Losartan treatment significantly restored Nox2, and slightly increased Nox4 compared to SHADR group, but Nox4 protein level was still significantly lower than in control. Single tempol or combined treatment showed no significant differences from SHADR group regarding Nox2 and Nox4 levels.

### 7. Immunoperoxidase staining

**Fig 9:**
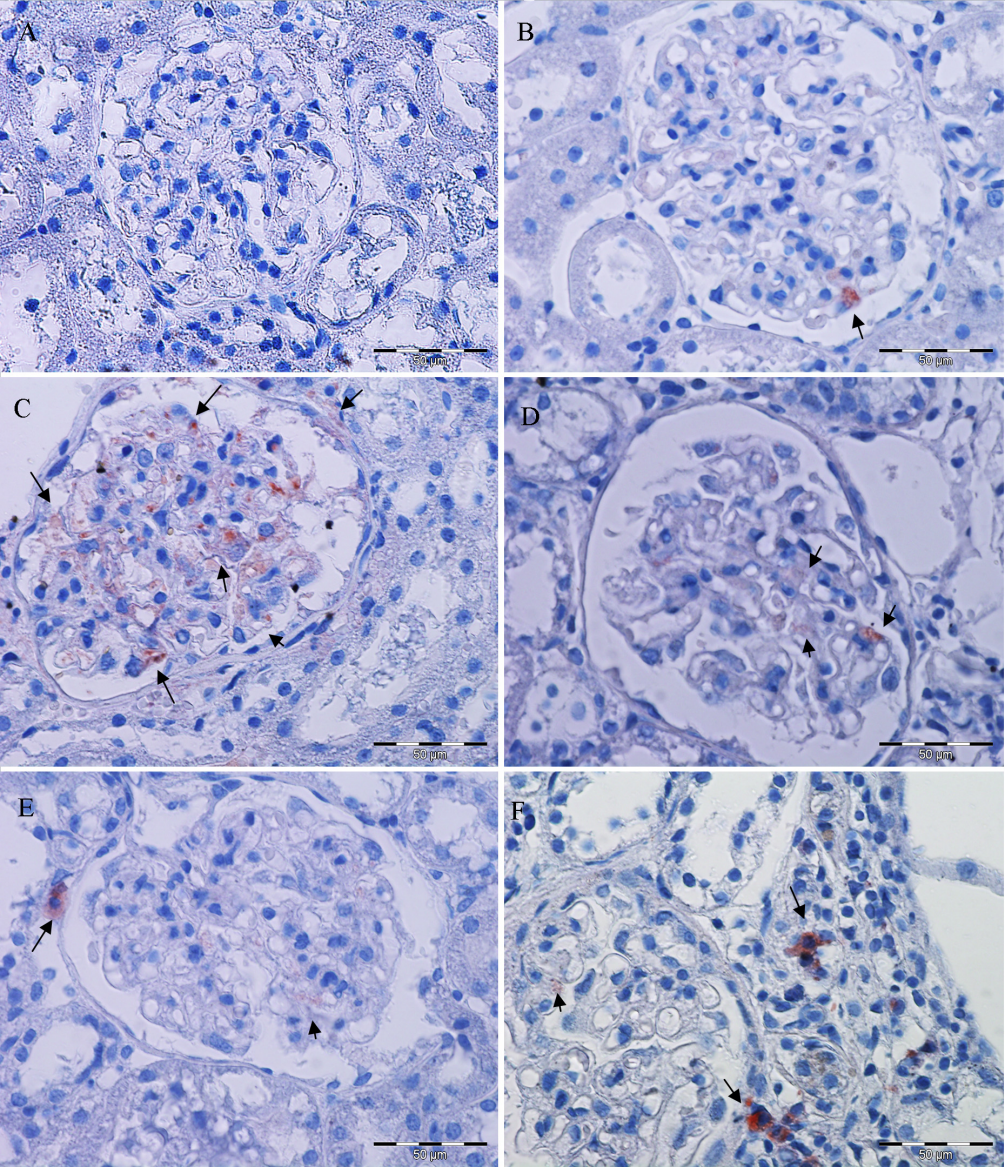
Glomerular nestin expression in experimental groups. (**A**) Negative control (×200). (**B**) SHC group: Nestin was observed focally in some of glomeruli, whereby expression was usually limited on single podocyte per glomerulus. (**C**) SHADR group: Diffuse glomerular nestin expression was detected involving almost all podocytes within glomerulus. After losartan and tempol treatment, either single or in combination, kidneys restored nestin expression similar to controls (SHC group). Thus, SHADR+L group (**D**), SHADR+T group (**E**) and SHADR+T+L group (**F**) exhibited similar glomerular nestin immunomorphological profile as observed in control animals. However, in addition to rare expression in podocytes, nestin was also detected in some interstitial cells, mainly within periglomerular area, as it is shown in images (E) and (F). Arrows indicate nestin expressing cells (podocytes and interstitiual cells). Magnification for images B-F ×400.

The localization of nestin positive staining in all experimental groups and negative control are shown on Fig 9. Nestin was observed focally in some of glomeruli from SHC group, whereby expression was usually limited on single podocyte per glomerulus (Fig 9B). In SHADR group diffuse glomerular nestin expression was detected involving almost all podocytes within glomerulus (Fig 9C). After losartan and tempol treatment, either single or in combination, kidneys restored nestin expression similar to control. Thus, SHADR+L group (Fig 9D), SHADR+T group (Fig 9E), and SHADR+T+L group (Fig 9F) exhibited similar glomerular nestin immunomorphological profile as observed in control animals. However, in addition to rare expression in podocytes, nestin was also detected in some interstitial cells, mainly within the periglomerular area, as it is shown in images Fig 9E and 9F.

### 8. Correlation Analysis of Parameters

The correlations among examined parameters (Table 1) demonstrate that Up/cr is positively correlated with pTBARS, as well as with parameters of morphological changes of glomeruli and tubules (sclerotic changes in glomeruli and index of tubular damage). In addition, sclerotic glomerular changes are strongly correlated with the index of tubular damage (r = 0.6269, p = 0.001), and these morphological changes of kidney showed significant positive correlation with Pcr. Further, significant positive correlation was observed between Up/cr and PCOs parameters (Table 1). SOD activity showed significant positive correlation with GSH-Px activity in erythrocytes (r = 0.4787, p = 0.021), and in kidney as well (r = 0.8036, p = 0.000).

**Table 1:** The correlation among Up/cr, Up, Pcr, pTBARS, PCOs, and morphological changes of the kidney in all experimental groups (n = 23).

	Up/cr (g/mmol)	Pcr (μmol/l)	pTBARS (nmol/ml)	PCOs (nmol/mg prot)	Sclerotic changes in glomeruli	Index of tubular changes
Up/cr (g/mmol)		r = 0.3091	**r = 0.4524**	**r = 0.4491**	**r = 0.5561**	**r = 0.5251**
		p = 0.151	***p = 0*.*030***	***p = 0*.*032***	***p = 0*.*006***	***p = 0*.*010***
Pcr (μmol/l)	r = 0.3091		r = 0.2896	r = -0.3461	**r = 0.5124**	**r = 0.5118**
	p = 0.151		p = 0.180	p = 0.106	***p = 0*.*012***	***p = 0*.*013***

Up/cr—urine protein-to-creatinine ratio, Pcr–plasma creatinine level, pTBARS–plasma TBARS level, PCOs–protein carbonyl content in kidney. Marked correlations are significant at *p* < 0.05; N = 23.

## Discussion

Rats that are given adriamycin provide an experimental model of the association between proteinuria and loss of renal function [21]. A cumulative dose of adriamycin 4 mg/kg used in the present study induced an early stage of proteinuric nephropathy with massive proteinuria and advanced glomerular sclerosis with capsular adhesion of glomerular tuft and tubulointerstitial lesions in SHR as previously reported [3,5], followed by a significant increase of plasma creatinine level. Moreover, abundant nitrite excretion was observed. In addition, the present study has for the first time shown overall kidney protein carboxylation, increased MMP-1 expression, intensive nestin immunostaining in glomeruli, and downregulation of Nox4 and Nox2 proteins in SHADR rats with the early stages of proteinuric nephropathy.

In this early phase of ADR nephropathy a decline of renal function was in correlation with glomerular and tubular injuries, and with the concurrent enhancement of systemic oxidative stress (increased TBARS production rate) and kidney protein oxidation. Yagmurca et al. also showed that single i.p. injection of ADR (20 mg/kg) in Sprague–Dawley rats caused significant enhancement of protein oxidation in the kidney [22]. Other studies found significant increases of lipid peroxidation in plasma, but no changes in antioxidant activities of SOD, CAT and GSH-Px in erythrocytes [23,24]. These results are in agreement with finding from the present study.

The present study has shown that chronic single treatments with SOD mimetic, tempol and AT1R blocker, losartan or their combination significantly enhanced plasma antioxidant capacity and erythrocyte antioxidant enzyme activities, thus reduced plasma lipid peroxidation, leading to the improvement of systemic oxidative status in SHR with the early course of ADR-induced nephropathy. These results are in accordance with previously obtained results applying olmesartan in 5/6 nephrectomy which was effective in reducing systemic oxidative stress [25], as well as with finding that tempol alone attenuated systemic oxidative stress in the model of L-NAME induced hypertension [26], while tempol alone or tempol plus enalapril significantly attenuated plasma oxidative stress in AngII-induced hypertensive rats [27]. Furthermore,^.^O_2_^-^ generation in the aorta was completely diminished after combined tempol-losartan treatment in renal hypertensive rats (2K1C), [14], and after tempol and its combination with enalapril, but not enalapril alone in AngII-induced vascular oxidative stress [27]. Interestingly, in our study, tempol in combination with losartan induced no further amelioration of systemic oxidative stress, and reduced SOD and CAT activities compared to single treatments in SHADR, probably as a result of attenuated^.^O_2_^-^ production in erythrocytes of SHADR+T+L rats.

Reactive oxygen species generation has been suggested to be responsible for ADR cardiotoxicity and nephrotoxicity. The alterations in renal function in SHR with ADR nephropathy, as noticed by the development of massive proteinuria in this study, were also accompanied by a significant reduction of SOD and GSH-Px activities in the kidney. The above findings are consistent with those previously reported in diabetic and ADR-induced nephropathy [11,28–30]. Because ADR accumulates mainly in the kidney [31], and given that the kTBARS remained unchanged after ADR treatment in the present study, similar to results obtained by Zima et al. in *Wistar* rats [32] or in BALC/c mice with early ADR nephropathy one week after application of 10mg/kg [28], we performed protein carbonyl test to find out whether protein carbonilation, an irreversible process induced *in vivo* by all types of reactive oxygen and nitrogen species including peroxynitrite, and followed by loss of enzymatic activities, loss of ligand binding properties, increased susceptibility to proteolytic activities, aggregation, and modification in the transcriptional activities [7], could participate in ADR-induced nephropathy in SHR. Increased PCOs in the kidney of SHADR group was associated with a significant reduction in the antioxidant enzyme activities of kSOD and kGSH-Px. These results were consistent with the findings described by others in diabetic rats [11], and ADR-induced nephropathy models [22,28–30]. Taking into account that peroxynitrite when acts as an oxidant produces nitrite and hydroxide ion rather than isomerizing to nitrate [33], we hypothesized that the increased nitrite production in the kidney of SHADR and the reduced level of nitrate that we observed compared to SHC, indicated an elevated level of peroxynitrite induced protein oxidation in this group, thus in conditions of reduced kidney antioxidant defense contributed to the deterioration of renal structure and function in this model of proteinuric nephropathy. Six-week losartan treatment reduced protein oxidation by the mechanisms involved elevation of kSOD and kGSH-Px activities and reduction of nitrite level, thus reducing peroxynitrite generation. Completely restored SOD and GSH-Px due to chronic AT1R blockade in our study were consistent with that previously reported [29,34].

Tempol has been shown to protect proteins from oxidative damage [9]. Administered to SHADR rats it also resulted in a reduction of protein carbonylation and nitrite production, although the activities of kSOD and kGSH-Px were reduced in this group, as in SHADR compared to SHC and SHADR+L. The similar antioxidant effect of tempol was also described by Sainz et al. in L-NAME hypertensive rats [35]. Tempol could directly scavenge superoxide anion and thus prevent the formation of hydrogen peroxide [36], a substrate for kGSH-Px, or could enhance the effects of NO by preventing its bioinactivation by^.^O_2_^-^ [9]. Nitrite production in the SHADR+T group was significantly reduced, while the nitrate level was elevated compared to SHADR, indicating the increase of biologically active NO and reduced NO that could act like peroxynitrite oxidant, resulting in prevention of protein oxidation. This notion is in accordance with previous reports [37] showing that tempol suppressed glomerular peroxide production, because it may easily permeate the podocytes and glomerular endothelial cells (glomerular basement membrane) during glomerular filtration due to its low molecular weight (172 Dalton). The improvement of oxidative defense in SHADR+T group was accompanied by almost completely abolished proteinuria, thus slowing the progression of glomerular and tubular damage. Interestingly, when used together losartan and tempol, almost complete recovery of SOD activity was found, but GSH-Px production still remained at the significantly lower level as in ADR-treated SHR. Likewise, in SHADR+L and SHADR+T groups NO_2_ excretion was depleted after combined treatment, but was not followed by an elevation of NO_3_ elimination from the kidney. An increase of SOD activity, which is responsible for enhancement of H_2_O_2_, and increased NOx productions in the kidney of SHR with ADR-nephropathy, could be counted for the failure of combined therapy to overcome single treatment. Although the reasons for such effects are not clear, we suppose that in this condition potentially damaging oxidants such as hydroxyl radical (OH^.^) could cause NO inactivation with concomitant peroxynitrite (ONOO^-^) formation, as previously demonstrated by McBride et al. [38], who showed that the reaction of NO, H_2_O_2_ and SOD may have pathological implications, especially under conditions with elevated NO and H_2_O_2_ levels. NO can bind spontaneously to thiol groups and decrease GSH-Px activity [39], therefore, contribute to increase of H_2_O_2_ production.

Peroxynitrite is a highly reactive cytotoxic compound capable of initiating lipid peroxidation, and nitration of tyrosine residues (nitrotyrosine), resulting in loss of protein structure and function, producing tissue injury and dysfunction [40]. Furthermore, cysteine oxidation by peroxynitrite may result in enzyme activation instead of inhibition, as demonstrated for matrix metalloproteinases (MMPs), which have been recently implicated as an important mechanism of peroxynitrite-dependent toxicity in heart disease and stroke [41]. We found MMP-1 to be elevated in the kidney of our proteinuric SHADR rats compared to control. The immunostaining that had been performed by Ahmed et al. [42] indicated a strong induction of MMP-1 within the glomerular basement membrane and mesangial matrix, but lack in tubules of 5/6 nephrectomized *Wistar* rats. Present results showed that tempol was most successful in improving ADR-induced MMP-1 overexpression, most likely due to prevention of peroxynitrite provoked deterioration of proteins.

Regarding the effect of all treatments on glomerular and tubular injury, obtained results showed that both, losartan and tempol, contrary to combined treatment, highly suppressed tubular damage, while tempol preserved both, glomeruli and tubules in SHR with the early course of ADR-induced nephropathy. Earlier studies in the same experimental model also demonstrated that chronic losartan treatment improved kidney structure [3], while tempol effectively suppressed glomerular and tubular damage in various models of proteinuric nephropathy [10–12]. Here, Up/cr ratio showed higher positive correlation with glomerulosclerosis, than with an index of tubular damage, and was also in accordance with the MMP-1 protein expression. Previous reports proposed that elevated extracellular matrix (ECM) proteolysis causes damage to the glomerular basement membrane and/or the mesangial matrix that may initiate remodeling by triggering the compensatory over production of ECM components resulting in both inappropriate and disorganized matrix [42]. Even though proteinuria, MMP-1, and sclerotic changes of the glomeruli were similar in SHADR+L and SHADR+T+L group, losartan expressed the most powerful protective effect on ADR-induced tubular injury, while combination therapy failed to alleviate damage induced by ADR. We suppose that adverse renal effects of combined therapy could be related to more complex mechanisms that involved the interaction between free radicals and AT1R, possibly affecting the ability of the kidney NO generation in this experimental model. Finally, the high correlation between Up/cr ratio and plasma lipid peroxidation pointed out the important role of systemic oxidative stress in the progression of ADR-induced nephropathy concomitant with hypertension.

The exact roles of NADPH oxidase–derived ROS and specific Nox isoforms in the kidney still remain unclear [43]. Nox4 contributes to the formation of kidney H_2_O_2_, while Nox2, a phagocytic NADPH oxidase originally described in neutrophils and macrophages [44], generates ROS in response to inflammation. The present study showed downregulation of both, kNox4 and kNox2 protein expression in ADR-induced proteinuric nephropathy model. These results are in agreement with previous findings that renal Nox4 expression, mainly localized to tubular cells, decreased in the course of diabetes and was not associated with a compensatory up regulation of Nox1 or Nox2 [45] in global and inducible Nox4 knockout mice. Contradictory to the established view that NADPH oxidases are the most important source of ROS production in the kidney [43], they suggested that under specific conditions Nox4 may even slightly limit injury and disease progression. Furthermore, Zhang et al. found that NADPH oxidase-derived ROS and redox-sensitive cell signaling pathways do not promote but rather limit lipopolysaccharide-induced acute inflammatory responses in gp91phox and p47phox knockout mice [44]. This opinion coincides with our observation obtained with losartan treatment because kNox4 was reduced in SHADR+L group, and losartan was able to significantly restore kNox2 protein expression. Together with previously mentioned benefits of losartan in our study, we suggested that this restoration of anti-inflammatory kNox2 signaling could contribute to mitigation of renal structural and functional changes induced by ADR.

In the present study the expression of nestin was found in some of glomeruli limited on single podocyte per glomerulus. Adriamycin induced diffuse distribution of nestin expression involving almost all podocytes. These data are in agreement with previously published results of up regulation of nestin, vimentin and desmin immuneexpression in puromycin aminonucleoside induced nephrosis in *Wistar* rats [46]. All treatments either single or combined restored nestin expression similar to controls.

In conclusion, all treatments reduced protein-to-creatinine ratio (marker of proteinuria), plasma TBARS production, kidney protein carbonylation, nitrite excretion, increased antioxidant capacity and restored kidneys nestin expression almost similar to control values. Both single treatments significantly improved systemic and kidney antioxidant defense, bioavailability of renal nitric oxide, reduced kMMP-1 protein expression and renal injury, thus retarded CKD progression. Losartan improved blood pressure, as well as tubular injury and restored anti-inflammatory defense by reverting kNox2 to the expression as in control. Interestingly, tempol was more successful in reducing systemic oxidative stress, proteinuria, kMMP-1 and glomerulosclerosis. Results from our study revealed that combined treatment with AT1R, losartan, and SOD mimetic/radical scavenger, tempol, failed to overcome the beneficial effects that they produced when applied alone in slowing down the progression of ADR-induced nephropathy in SHR. Further studies will be necessary to elucidate the exact mechanisms responsible for their interaction and effects on the progression of chronic kidney disease with hypertension.
